# Integrative Rehabilitation for War-Related Polytrauma: A Narrative Review of Multidimensional Strategies and Implementation Challenges

**DOI:** 10.3390/healthcare14172778

**Published:** 2026-09-01

**Authors:** Ji Sun, Y. M. R. C. Hirushan, Harith Randula, Weixin Zhang, Qianhao Wu, Jia Han

**Affiliations:** 1 Jiangwan Hospital of Hongkou District, Shanghai University of Medicine and Health Sciences, Shanghai 201318, Chinapmc.ravinduc24@gzu.edu.cn (Y.M.R.C.H.);; 2 State Key Laboratory of Green Pesticide, Guizhou University, Huaxi District, Guiyang 550025, China; 3 College of Rehabilitation Sciences, Shanghai University of Medicine and Health Sciences, Shanghai 201318, China

**Keywords:** rehabilitation, polytrauma, post-traumatic stress disorders, phantom limb, robotics

## Abstract

Objective: Modern high-intensity warfare, such as the Russo-Ukrainian conflict, generates a high incidence of multisystem polytrauma, blast-induced traumatic brain injury, and limb amputations often complicated by post-traumatic stress disorder and chronic pain. Traditional specialised rehabilitation protocols are structurally inadequate for these co-occurring symptoms and rely heavily on medical treatments, increasing the risk of opioid dependency. The primary aim of this review is to conceptualise a multidimensional integrative framework for war-related polytrauma; a secondary aim is to evaluate and summarise the evidence underpinning its key rehabilitation strategies for limited-resource, post-conflict environments. Methods: This narrative review synthesises evidence identified through targeted database searches and purposive selection guided by clinical relevance, methodological quality, and applicability to war-related polytrauma across physical, technological, and non-pharmacological rehabilitation domains. Evidence was evaluated using a four-tier (A–D) hierarchy, comprising Tier A (systematic reviews and RCTs), Tier B (prospective cohort studies and non-randomised controlled trials), Tier C (descriptive, case-series, and retrospective studies), and Tier D (exploratory, mechanism-based, or expert opinion evidence). No systematic inclusion/exclusion protocol was applied, and no claims of comprehensive search coverage are made. Results: Concurrent physical and trauma-focused psychological rehabilitation, evidenced by reduced pain and psychological symptom burden alongside improved functional independence, is supported by high-quality evidence (Tier A) for managing comorbid polytrauma. Robotic exoskeletons and AI-supported tele-rehabilitation demonstrate dose-dependent motor benefits in selected rehabilitation populations (Tier A–B), but their scalability in wartime settings is contingent on electricity supply, connectivity, equipment maintenance, and trained personnel. Acupuncture shows Tier A evidence for neuropathic pain and Tier B cost-effectiveness; evidence for phantom limb pain and PTSD is preliminary (Tier C–D) and requires dedicated RCTs in veteran populations. Ayurvedic herbal interventions are exploratory (Tier C–D) with no war-specific clinical trials; mechanistic plausibility is discussed as hypothesis-generating only. Conclusions: Addressing the war-related rehabilitation gap requires integration of biomedical care, technology-assisted rehabilitation, and evidence-graded non-pharmacological adjuncts within a structured biopsychosocial framework. Future priorities include pragmatic, adapted trial designs (e.g., stepped-wedge or cohort-embedded designs, which are more feasible than classical RCTs under wartime conditions) for acupuncture in phantom limb pain, feasibility trials for robotic rehabilitation in frontline-adjacent facilities, and health-economic modelling adapted to the Ukrainian context.

## 1. Introduction

Modern high-intensity warfare has shifted the rehabilitation challenge from survival alone to the long-term management of complex, interacting disabilities. The ongoing Russo-Ukrainian conflict, one of the most intense interstate conflicts in recent decades, illustrates this shift. High-energy blast and penetrating mechanisms have produced lumbopelvic dissociation, penetrating traumatic brain injury (TBI), multiple-limb amputations, and blast-related multisystem polytrauma at rates far exceeding those reported in many previous conflicts [1,2]. Congested airspace, mobile front lines, and damaged infrastructure have prolonged evacuation times compared with the wars in Iraq and Afghanistan. This has increased the duration of care under critical conditions and contributed to the survival of patients with injury patterns that were previously often fatal [1,3]. Although most combat deaths still occur before medical treatment, predominantly from uncontrolled haemorrhage, survivors who reach definitive care frequently present with combined extremity trauma, blast-induced TBI, spinal injury, and other multisystem lesions [1,2,4]. These injury profiles require rehabilitation models that address concurrent physical, neurological, psychological, and social disability rather than sequential single-system recovery.

The expected disability burden is substantial. By moderate estimates drawn from qualitative needs-assessment reporting, more than 1.2 million Ukrainian veterans are projected to eventually require long-term support after major combat operations. This figure represents a forward-looking projection of future caseloads rather than a current registered count. Because the number of wounded far exceeds the number of deaths, this population places demands on the rehabilitation system that acute surgical frameworks cannot meet [1,3]. Recent population surveys report post-traumatic stress disorder (PTSD) in 25.9% and complex PTSD (CPTSD) in 14.6% of the general population, with significantly higher rates in areas near the front lines and among women [5]. Among veterans, structured psychological screening at admission to rehabilitation reveals severe symptoms of PTSD (39%) and signs of suicidal behaviour (7%) [6]. The co-occurrence of TBI, PTSD, and chronic pain represents a defining disability pattern of modern warfare. These conditions interact bidirectionally: pain worsens psychological symptoms, PTSD amplifies pain sensitivity and reduces coping capacity, and psychological dysfunction reduces compliance with physical rehabilitation [7].

Traditional rehabilitation models, primarily based on civilian single-diagnosis models, are poorly matched to this. Care is often disjointed, with physical, psychological, and social aspects of care occurring in isolation [8]. Chronic pain, particularly phantom limb pain (PLP) and neuropathic pain, affects 57–72% of military amputees [9]. Management of these conditions remains dominated by pharmacological interventions. While offering short-term relief, these medications risk opioid dependency in a population already vulnerable to addiction due to comorbid PTSD and TBI [9,10]. Current U.S. Veterans Affairs/Department of Defense (VA/DoD) and Centers for Disease Control and Prevention (CDC) guidelines explicitly discourage routine long-term use of opioids in this group, favouring non-opioid approaches and multimodal treatment [10,11,12]. The psychological factors of disability catastrophising, fear-avoidance, moral injury, and occupational identity loss remain under-recognised within biomedical models of recovery that equate it with physical repair [13].

**Figure 1:**
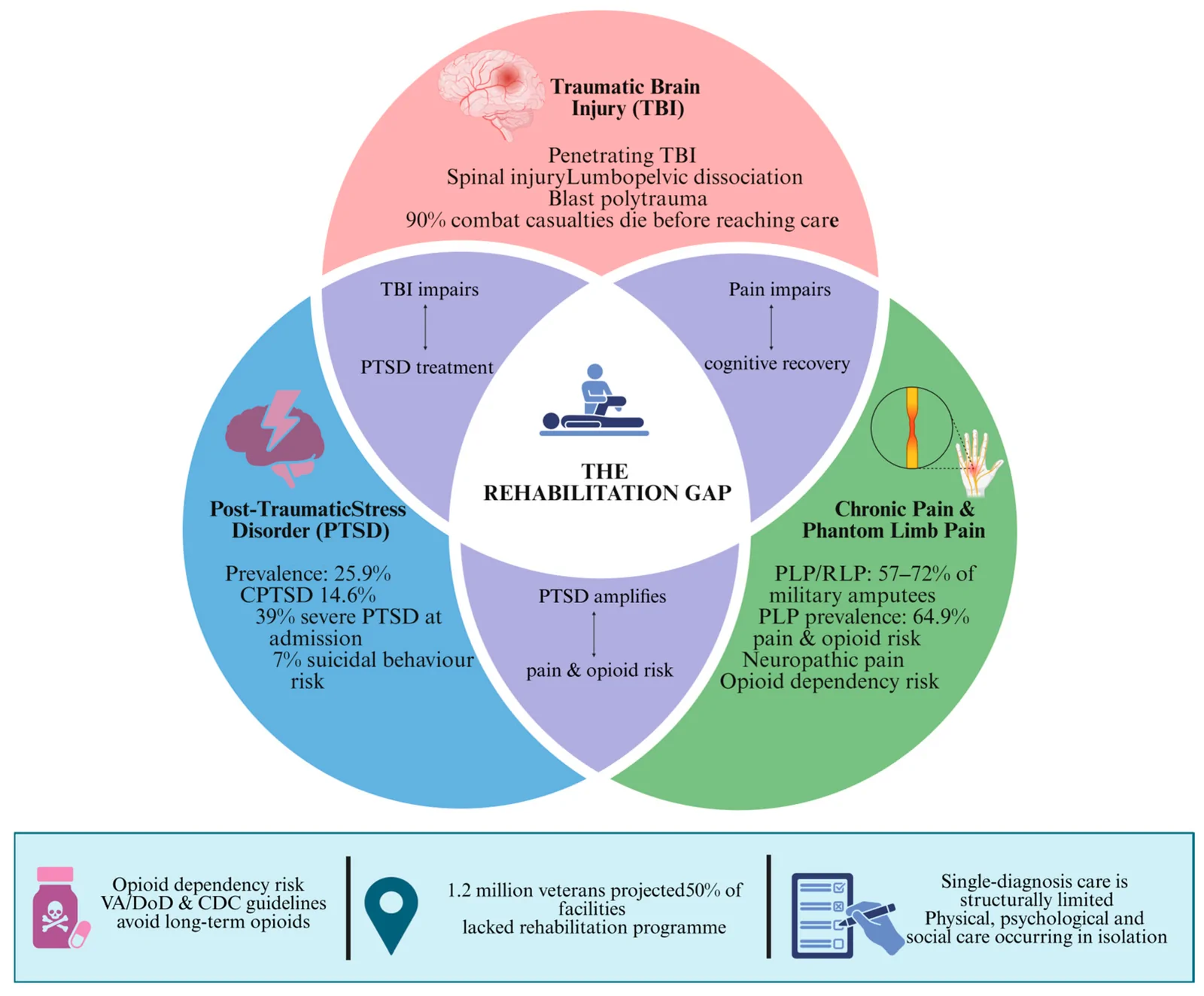
The “trinity of wounds” and the multidimensional rehabilitation gap in modern warfare. Bidirectional arrows indicate evidence-based amplification pathways between TBI, post-traumatic stress disorder (PTSD), and chronic pain. The central zone represents the rehabilitation gap produced when traditional single-domain care addresses only one domain in isolation [5,7,9,13].

These challenges are evident in Ukraine’s rehabilitation system, even with legislative reform in 2020. Real-time qualitative evaluation revealed that only 50% of facilities had rehabilitation programmes; electronic medical record systems were not integrated between acute care and rehabilitation in 47% of cases [1]. Mental health services were highly fragmented within psychiatric departments despite reported distress in up to 95% of military personnel, and no effective national rehabilitation strategy existed to guide system-wide response [1]. The gap between the multidimensional physical, psychological, social, and existential needs of people affected by war and the disjointed, largely biomedical systems available to support them is defined in this review as the rehabilitation gap (Figure 1).

This review addresses that gap by synthesising evidence across biomedical rehabilitation, technology-assisted rehabilitation, and evidence-graded non-pharmacological adjuncts for war-related polytrauma. Unlike reviews that examine individual rehabilitation modalities in isolation, this article applies a three-tiered (I-III) implementation framework stratified by resource availability, injury profile, and systemic constraints in the Ukrainian context. Each intervention is assigned an explicit evidence tier to clarify the strength and directness of available support across heterogeneous rehabilitation domains. Given that Ukraine is the primary focus, the evidence is not assumed to be universally transferable across all conflict settings. Differences in health-system capacity, conflict dynamics, evacuation logistics, and population demographics limit direct generalisation. Therefore, this review draws on international evidence where war-specific data are scarce while maintaining a specific focus on the rehabilitation needs generated by the current Ukrainian conflict. The proposed contribution is therefore an explicit conceptual architecture that maps established interventions to recovery domains, evidence tiers, and resource-stratified stages; it is not presented as a de novo causal theory or an empirically validated integrated-care model.

## 2. Method

This paper is a narrative review designed to synthesise clinically relevant and implementation-focused evidence for war-related polytrauma rehabilitation. The review was prepared through purposive literature searches conducted in PubMed, Scopus, Cochrane Library, Embase, and Google Scholar between January 2024 and April 2026, using terms including “war polytrauma rehabilitation”, “blast injury rehabilitation”, “phantom limb pain veterans”, “traumatic brain injury military”, “robotic rehabilitation amputee”, “acupuncture neuropathic pain”, “Tai Chi PTSD veterans”, “osseointegration military”, “moral injury treatment”, and “telerehabilitation conflict settings”. Reference lists of retrieved articles were hand-searched for additional relevant sources. The final selection of included literature was guided by clinical relevance to the Ukrainian conflict context, evidence quality, and topical coverage as judged by the authors. This purposive, author-guided selection approach, while appropriate for a practice-oriented narrative synthesis, carries an inherent risk of selection bias: studies aligning with the authors’ prior clinical assumptions or with the proposed framework may have been more readily identified or emphasised than disconfirming evidence. Because no systematic screening protocol or independent dual-reviewer selection was applied, this review should be interpreted as a practice-oriented narrative synthesis rather than a bias-minimised systematic assessment. The synthesis was structured to support a practice-oriented appraisal across heterogeneous rehabilitation domains rather than a quantitative meta-analysis.

All included evidence was graded using the Oxford Centre for Evidence-Based Medicine (OCEBM) 2011 Levels of Evidence framework [14], adapted for rehabilitation contexts into a four-tier operational hierarchy: Tier A (systematic reviews and RCTs; OCEBM Level 1–2), Tier B (prospective cohort studies and non-randomised controlled trials; OCEBM Level 3), Tier C (descriptive, case-series, and retrospective studies; OCEBM Level 4), and Tier D (exploratory, mechanism-based, or expert opinion evidence; OCEBM Level 5). Tier assignments were reviewed and agreed upon by the authors and are reported throughout the review to indicate the relative strength, directness, and implementation relevance of the available evidence. Where authors’ initial tier assignments diverged, the disagreement was resolved through discussion of the underlying study design and applicability to the war-polytrauma context until consensus was reached; no formal inter-rater statistic was calculated, consistent with the narrative (non-systematic) nature of this review. Because the same author judgement also shaped the relative emphasis and interpretation of findings, consensus reduced overt inconsistency but did not eliminate interpretive subjectivity or confirmation bias. Where the underlying evidence originated from non-war populations (e.g., civilian stroke, spinal cord injury, or chronic pain cohorts), the tier reflects the methodological quality of the source study, not the directness of its applicability to war-related polytrauma; directness of applicability is instead addressed narratively at each point of use and summarised in the interpretive limitations below.

This review focuses on adult military and civilian war-affected populations of working age. Paediatric and geriatric rehabilitation populations, while acknowledged as important in the Ukrainian conflict context [15], are noted where relevant but are not the primary focus of the present synthesis.

## 3. Injury Burden and the Rehabilitation Gap

For the purposes of this review, polytrauma is defined according to the Berlin Definition [16] as the presence of an Abbreviated Injury Scale (AIS) score of ≥3 in two or more distinct body regions, in combination with at least one of five physiological derangements, including haemodynamic instability (systolic blood pressure ≤ 90 mmHg), loss of consciousness (Glasgow Coma Scale ≤ 8), metabolic acidosis (base excess ≤ −6.0), coagulopathy (international normalised ratio ≥ 1.4), or age ≥ 70 years [17]. This definition replaces the older ISS ≥ 16 threshold because it accounts for bodily resilience. Furthermore, it is more closely aligned with survival prediction and recovery trajectories in severely injured patients [18]. The U.S. Department of Veterans Affairs definition—traumatic injuries to two or more body systems with at least one life-threatening—is used interchangeably in cited sources and encompasses the same clinical population [19]. This definitional clarity is important because war-related survivors rarely present with isolated impairments; rather, they enter rehabilitation with overlapping physical, neurological, psychological, and social needs that exceed the scope of single-diagnosis pathways.

### 3.1. Physical Injury Profiles

The Russo-Ukrainian conflict has generated injuries characterised by blast mechanisms, often with combinations of penetrating, blunt, and thermal injuries. Fractures and amputations from high-energy explosions and thermobaric weapons mandate staged debridement, external fixation, and amputation decisions with limited resources [1]. Lumbopelvic dissociation, once rare, now occurs at markedly higher rates. This increase is a consequence of both the destructive power of modern weaponry and improved frontline haemorrhage control, which enables more severely injured patients to reach surgical care [1]. Penetrating TBI caused by shrapnel and blast-wave exposure has also become more prominent as the conflict has escalated. Neurosurgical innovation including liberal computed tomography (CT) scanning, tele-neurosurgical consults, and early surgical decompression has led to better outcomes, but highlighted the importance of early imaging and stabilisation [4]. Polytrauma, defined as multiple simultaneous life-threatening injuries, is the major cause of disability in young adults [15]. The combination of TBI, orthopaedic, and visceral injuries presents complexity that outstrips the capacity for single-system, sequential care [3,20,21]. Table S1 maps these injury categories to targeted rehabilitation strategies and ranks them by evidence strength and implementation priority [1,2,4,6,20,21,22,23,24,25,26,27,28,29,30,31,32,33,34,35,36,37,38,39,40,41,42,43,44,45,46,47,48,49,50,51,52,53].

### 3.2. Psychological Burden

The conflict has had profound psychological effects. Comparative analysis with other nations found that Ukrainian respondents had significantly greater levels of psychological distress than Polish or Taiwanese respondents assessed at the same time [54]. In a sample of displaced people, 93.5% experienced acute stress disorder (ASD) [55]. Severity was associated with subsequent PTSD [55]. Older adults in de-occupied villages reported PTSD prevalence of 47–51% with comorbidities of chronic illness and loneliness [56,57].

Neurological injury and chronic pain frequently coexist after blast exposure. Pain after mild TBI negatively affects function, mood, and sleep, generating an interconnected neuro-pain disability profile that is particularly relevant to military populations exposed to blast mechanisms [7,58,59]. Phantom limb pain (PLP) and residual limb pain (RLP) affect 57–72% of military amputees and are commonly associated with PTSD, depression, chronic opioid use, and reduced prosthetic function [9,22]. Meta-analytic synthesis supports the prevalence of PLP at 64.9% in amputations, with upper-limb amputations and time since amputation as prominent risk factors, highlighting the need for early pain intervention in early rehabilitation [23,24,25].

### 3.3. Ukraine’s Rehabilitation System Is Under-Resourced

The Ukraine rehabilitation system is dramatically under-capacitated to manage its caseload. Staffing levels are critically low. There are not enough trained rehabilitation physicians, physiotherapists, prosthetists, psychologists, and social workers, and the damage to healthcare infrastructure and loss of personnel in the east have worsened these challenges [1]. The predominant model in many institutions, which combines remnants of Soviet-style sanatorium practice with under-resourced active rehabilitation, creates a barrier to the psychosocially complex, patient-centred practice of evidence-based rehabilitation [1]. Healthcare is inequitably distributed, with delays for patients and a dysfunctional national reintegration plan to manage the handover from acute care and reintegration [8].

The risk of opioid dependency, intensified by co-morbid PTSD, represents the most pressing pharmacological challenge in managing complex chronic pain [60,61]. The failure to prioritise multidisciplinary, non-chemical pain management creates a critical risk that Ukraine will repeat the high rates of addiction observed in Western veterans [10,12]. Taken together, these physical, psychological, and system-level pressures define the practical rehabilitation gap: a growing population of war-injured survivors whose needs are multidimensional, while available care pathways remain fragmented, unevenly resourced, and insufficiently integrated across the continuum from acute stabilisation to long-term reintegration.

## 4. Core Biomedical Rehabilitation: Evidence and Limitations

### 4.1. Western Polytrauma Rehabilitation Protocols

The U.S. Veterans Affairs (VA) Polytrauma System of Care, which represents the best documented system for military polytrauma rehabilitation, takes a hierarchical approach ranging from the acute phase through to polytrauma transitional rehabilitation programmes (PTRPs) and community reintegration. Multidisciplinary teams (physiatrists, occupational therapists, neuropsychologists, speech-language pathologists, prosthetists) simultaneously target physical, cognitive, and psychological dysfunction. These teams provide 5–6 h of daily therapy, accompanied by structured reintegration into the community [62,63]. Outcomes in terms of the Functional Independence Measure show clinically relevant improvements in cognitive and physical function, although heterogeneity in patient profiles and attrition should be considered when interpreting long-term outcomes [64].

Osseointegration, in which a titanium implant anchors the prosthesis directly to bone, is an established reconstructive option for selected above- and below-knee amputees. A 10-year prospective cohort study of 51 transfemoral amputees treated with the OPRA system reported sustained improvements in mobility and quality of life across the Q-TFA and SF-36 instruments throughout the follow-up period. Swedish OPRA programme data report a 92% implant success rate at five years with sustained benefits at 15-year follow-up, with patients reporting increased prosthesis wear time, improved function in above-knee amputees, and reduced pain. UK military-specific data confirm successful outcomes in blast-injured personnel, with the importance of multidisciplinary pain management during the osseointegration rehabilitation phase emphasised [42]. Taken together, these data support a Tier B assignment for osseointegration in selected transfemoral amputees with blast-related limb loss. However, its implementation requires surgical expertise, infection-control capacity, long-term rehabilitation commitment, and careful multidisciplinary pain management, making specialist-centre delivery the most realistic pathway in the Ukrainian context.

The Acute Polytrauma Rehabilitation (APR) model within the UK’s Major Trauma Network emphasises the necessity of early intervention (0–72 h post-injury), ensuring that multidisciplinary support starts during the acute phase (Tier A) [20]. National audit data indicate that timely specialist rehabilitation is associated with mean net lifetime savings of approximately £500,000 per patient among those with very severe injuries, reflecting avoided nursing home placement, reduced long-term care utilisation, and restored economic productivity [20]. Veterans Affairs (VA) Intensive Evaluation and Treatment Programmes (IETPs) for chronic mild TBI provide specialised interdisciplinary care for persistent cognitive deficits, chronic pain, and comorbid PTSD, resulting in improved functional independence in similarly afflicted populations [65,66]. These models collectively show that early, coordinated, interdisciplinary rehabilitation can improve function, support reintegration, and reduce downstream care burden when adequate specialist capacity is available [15].

### 4.2. Critical Bottlenecks in the Ukrainian Context

Bottlenecks to implementing Western rehabilitation programs in Ukraine are structural, cultural, and pharmacodynamics-related. Lack of staff is compounded by damaged infrastructure. Cultural and linguistic challenges arise in war-injured patients evacuated for rehabilitation overseas that must be addressed through targeted intervention. A mixed-methods study of Ukrainian MEDEVAC patients receiving rehabilitation in a Norwegian institution found that patients initially expected a paternalistic healthcare model, in which physicians issue orders confirmed by other staff [8]. Consequently, these patients were troubled by questions concerning therapy goals and the preference of passive interventions, such as massage, over active rehabilitation [8]. Such concerns reflect Soviet-model medical epistemology. Patients with appropriate cultural mediation were ultimately satisfied (CSQ-8 median 30/32) [8], suggesting that this barrier can be overcome with appropriate support, a lesson for the domestic introduction of unfamiliar integrative therapies for Ukrainian military patients.

The psychological rehabilitation bottleneck is also important. The Invincibility Programme, a structured seven-day psychosocial rehabilitation module delivered to more than 3000 Ukrainian military personnel at a frontline-adjacent centre in Kharkiv, demonstrated that biopsychosocial rehabilitation can be both feasible and effective within 30–40 km of active combat operations [6]. Seventy-eight percent improved on all 16 negative psychological variables, 89% improved on more than half, and 96% viewed the program as necessary and important [6]. This operational experience provides proof of concept for integrated psychosocial rehabilitation under active wartime conditions and offers a practical platform onto which technology-assisted and adjunctive interventions may be layered.

## 5. Technology-Assisted Rehabilitation

### 5.1. Robotic Exoskeletons

Moderate-to-high-quality evidence supports robotic-exoskeleton-assisted gait training in stroke and spinal cord injury (SCI) (Tier A), with the magnitude of effects heavily dependent on the timing, intensity, and severity of injury. Pooled analyses in stroke show impressive improvements in walking speed, independence, and balance [26]. Effects are greatest in subacute patients undergoing 45–60 min per session and more than three hours per week of training, establishing a dose–response relationship directly relevant to rehabilitation planning [26]. In SCI, meta-analyses demonstrate clinically substantial improvements in the 6 min walk test, 10 m walk test, and timed up-and-go, with greater effects when training begins within the first six months post-injury [27]. RCTs support activity-dependent neuroplastic reorganisation, rather than mechanical compensation, as the main source of benefit [28,29,30].

Modular exoskeletons that only target the hips show identical gait improvements with lower device complexity and cost compared to full-leg designs, and are a scalable design for under-resourced clinical settings [67]. Chinese research institutions are among the most prolific contributors to exoskeleton development, reflecting technological consolidation and manufacturing scalability that may position domestically produced systems as a cost-effective procurement option for resource-constrained settings [68]. Budget modelling indicates that introduction of robotic exoskeletons may be cost-neutral due to reduction in therapist workload and secondary injuries [69]. Cost–utility modelling suggests incremental cost-effectiveness ratios of around USD $12,353 per quality-adjusted life year (QALY) for complete SCI within internationally established willingness-to-pay thresholds at the modelled cost levels [70]. The capital cost of the device is supported by labour cost savings when adoption rates reach the break-even point; thus, workforce training and patient throughput are critical in addition to procurement [69].

### 5.2. Brain–Computer Interfaces (BCIs)

BCIs complement robotic training by integrating brain intention into motor learning, establishing closed loops between the decoded neural activity and robotic motor output and sensory feedback. In chronic SCI, lower-limb robotics combined with electroencephalography (EEG)-based BCI showed clinically significant improvements in muscle strength and sensation after 3–7 weeks in motor-complete cases, which may be due to the re-engagement of spared neural structures (Tier B–C) [31,32]. In stroke, meta-synthesis suggests that MI-BCI positively impacts activities of daily living and upper-limb motor function when used in conjunction with conventional therapy for 4 weeks or more [33]. MI-BCI without conventional therapy does not necessarily surpass structured motor imagery (MI), confirming it as an adjunct to conventional therapy. Non-invasive EEG-BCI is generally well-tolerated, but the safety implications of protocol variability, unknown sustained effects, and technology need to be considered. BCIs should be considered as an intensification strategy within robotic or functional electrical stimulation protocols, especially for chronic or severe neurological injuries (Tier D for war-specific groups) [34].

### 5.3. Tele-Rehabilitation and AI-Augmented Delivery

The effectiveness of tele-rehabilitation relates to organisation, intensity, and feedback rather than distance. Meta-analysis shows that remote stroke care with maintained training dose and therapist interaction is as effective as centre-based care [71,72,73]. Minimal gain is noted with lightly supervised self-paced training (Tier A) [74]. This suggests tiered hybrid models rather than complete replacement of in-person care.

AI (artificial intelligence)-driven tele-rehabilitation has superior outcomes to passive tele-delivery. In chronic non-specific low back pain, AI-guided multimodal exercise achieved earlier and greater reductions in pain and disability than pre-recorded video instruction, attributable to real-time motion correction and adaptive progression [35]. Network meta-analysis synthesising 33 RCTs across 13 intervention categories confirmed that interactive, feedback-rich modalities, therapeutic exergaming (SUCRA = 87.6%) and robotic systems (SUCRA = 86.3%), consistently outperformed conventional care (SUCRA = 12.0–17.1%) and asynchronous tele-programmes across pain, functional, and range-of-motion outcomes [36]. These findings indicate that the critical distinction is not whether rehabilitation is delivered remotely, but whether remote delivery preserves therapeutic intensity, feedback quality, and progression control.

A home-based tele-robotic system with therapist-provided haptic feedback and patient-controlled surface electromyography (sEMG) achieved stable bidirectional tele-operation in feasibility testing [37,75,76], demonstrating technical feasibility under controlled conditions rather than established deployability in frontline-adjacent Ukraine. Initial trials of augmented-reality mirror therapy for phantom limb pain [38,39], however, showed good usability but poor adherence, illustrating the dangers of technological optimism without rigorous implementation planning [40]. Accordingly, technology-assisted rehabilitation should be evaluated not only by device efficacy, but also by training dose, user adherence, therapist workload, maintenance capacity, and integration into existing rehabilitation pathways.

Deployment under wartime conditions must also account for unreliable electricity and internet connectivity, interruptions in equipment maintenance and spare-parts supply, and limited opportunities for staff training. As an implementation scenario proposed by this review, a minimally viable Tier II (Sub-Acute and Specialist Rehabilitation/Chinese Intelligent Rehabilitation) facility would require backup power, low-bandwidth connectivity, equipment capable of local stand-alone operation, access to essential consumables and first-line maintenance, and staff trained in safety checks and basic troubleshooting. A hub-and-spoke model could reserve bandwidth-intensive, synchronous robotic, or AI-assisted sessions for better-equipped regional centres, while peripheral facilities use offline data capture and store-and-forward clinical review during connectivity interruptions. These asynchronous functions should be used to maintain monitoring and treatment continuity rather than be regarded as clinically equivalent substitutes for interactive, feedback-rich tele-rehabilitation.

## 6. Adjunct Non-Pharmacological Therapies

The therapies reviewed in this section are adjunctive interventions intended to complement, rather than replace, biomedical rehabilitation. They are considered here because they may contribute to symptom modulation, particularly chronic pain and psychological distress, and may help reduce reliance on pharmacotherapy in populations at elevated risk of opioid misuse. The quality of evidence across therapies varies greatly and is clearly outlined. Accordingly, these interventions are discussed according to their evidence strength, clinical relevance, and feasibility as adjuncts within a multidimensional rehabilitation pathway.

### 6.1. Acupuncture: Mechanisms and Clinical Evidence

Acupuncture’s analgesic mechanisms are described at a number of levels. At the periphery, mechanical stimulation by the needle activates dense afferent fibres (A-delta fibres at the muscle layer and C fibres at the skin layer). Local biochemical reactions are mediated by mechanosensitive ion channels (transient receptor potential (TRP), acid-sensing ion channel 3 (ASIC3)) that regulate pain via the spinal/supraspinal levels [41]. Local microenvironmental changes at the acupoint excite mast cells and macrophages via release of adenosine triphosphate (ATP), adenosine, cytokines, and chemokines, which further enhance the analgesic effect [77]. At the centre, acupuncture activates the mesolimbic reward system, by regulating dopamine levels in the areas responsible for pain and emotional processing [78]. Meta-analysis of RCTs indicates that acupuncture can reduce neuropathic pain severity, with effects plausibly mediated through peripheral nerve modulation and spinal gating mechanisms (Tier A) [42].

In preliminary studies, for the combat amputation population, mirror acupuncture on the contralateral side has been found to reduce PLP in lower-limb amputees, with proposed mechanisms of bilateral cortical remapping and activation of descending pain inhibitory pathways (Tier C–D) [43]. This approach should be investigated further in veterans in an RCT. According to economic data, pharmacopuncture may be cost-effective relative to physiotherapy for chronic low back pain, under the assumptions of a 3-year Markov model [44]. Traditional acupuncture for pain and mental health symptoms had an incremental cost-effectiveness ratio (ICER) of CAD $9030–10,766 per QALY in a Canadian program (within the willingness-to-pay range), with mean annual savings of CAD $3371 per patient from reduced prescription costs and specialist referrals (Tier B) [45]. Although these economic findings derive from non-war settings, they support the broader feasibility of acupuncture as a low-technology adjunct for pain management when appropriately regulated and integrated into rehabilitation care.

### 6.2. Traditional Chinese Medicine: Tai Chi and Mind–Body

Tai chi and qigong provide a dual physical and psychological approach to combat-injury rehabilitation, particularly in patients requiring balance restoration after blast trauma or prosthetic use and in those experiencing PTSD-related symptoms. Physically, tai chi results in gains in balance measures as well as improved anticipatory neuromuscular control via combining senses and movement [46]. Direct comparison with conventional vestibular rehabilitation demonstrated tai chi’s superior effects on gait biomechanics, particularly through functional reorganisation of lower-limb joint energetics rather than compensatory rigidity [47]. Long-term practitioners demonstrate enhanced proprioceptive processing and reduced reliance on vestibular input, a compensation mechanism likely to be clinically significant in patients with blast-induced vestibular injury [47].

Psychologically, a feasibility study with veterans had high enrolment and participation, with reduced PTSD symptoms, anxiety, and physical tension [48]. A current RCT is investigating the effectiveness of remote delivery through videoconferencing of tai chi for PTSD in veterans [79]. This may be an option in the geographically dispersed Ukrainian military. Integrating tai chi, yoga, acupuncture, and mindfulness, multimodal complementary and integrative health (CIH) interventions produced clinically meaningful reductions (effect size, d = −0.90) in PTSD symptoms alongside improvements in depression, pain, and psychological quality of life in veterans (Tier B) [49,50]. These findings support tai chi, qigong, and related mind–body approaches as clinically relevant adjuncts for balance, symptom regulation, and engagement in long-term rehabilitation, while reinforcing the need for structured delivery and follow-up in military populations. Structured tai chi or qigong delivery should not be assumed to be cost-free. Although these interventions are not inherently subscription-based, their implementation costs vary according to the delivery model and may include instructor training and supervision, staff time, venue provision, or digital-platform and subscription fees for remotely delivered programmes. Lower-cost approaches, such as group-based sessions or delivery by appropriately trained peer facilitators, may be feasible in some settings, but their safety, fidelity, and sustainability require local evaluation. Dedicated cost-effectiveness evidence for tai chi or qigong in war-related rehabilitation was not identified in the reviewed literature.

### 6.3. Mechanistically Plausible Phytotherapeutic Adjuncts for Combat-Injury Sequelae: A Hypothesis-Generating Review

Ayurvedic and related phytotherapeutic agents are considered here only as mechanistically plausible, hypothesis-generating adjuncts for inflammatory and psychological sequelae that may occur after combat polytrauma. Evidence from non-war populations suggests that *Withania somnifera* may reduce stress and anxiety symptoms, whereas curcumin and *Boswellia serrata* may have anti-inflammatory or anti-musculoskeletal-pain effects [80,81,82,83]. However, no war-specific clinical trials were identified, and heterogeneity in populations, preparations, dosing, and outcome measures prevents direct translation to combat-injured patients.

These interventions are therefore classified as Tier C–D and should not be interpreted as established components of war-related rehabilitation. Any future evaluation would require standardised preparations, systematic screening for herb–drug interactions, and safety monitoring in patients receiving anticoagulants or other post-trauma pharmacotherapy. Detailed botanical profiles, proposed mechanisms, safety considerations, and supporting evidence are provided in Supplementary Table S3 [51,52,53,80,81,82,83,84,85,86,87,88,89,90].

### 6.4. Reducing Pharmaceutical Burden: The Integrated Case

Across the adjunct therapies studied, a common policy-relevant theme emerges that adjuncts may function together in reducing the use of opioids and psychotropic medications to manage chronic pain and PTSD. Opioid use and misuse in veterans are not uncommon, where comorbid PTSD and TBI significantly elevate vulnerability to substance use disorder and self-harm [10]. Multicomponent exercise (aerobic, resistance, yoga) resulted in a large effect size (d = −0.90) reduction in PTSD symptoms without medication use [49]. A systematic review of 15 controlled studies of mind–body therapies reported reduced PTSD symptoms and retention rates of 70% or higher [91]. CIH delivered by tele-health significantly decreased the interference of pain in mood and sleep in veterans with co-occurring PTSD and pain [92]. Together, these studies support the inclusion of non-opioid, non-pharmacological strategies within rehabilitation models for war-related polytrauma, particularly when chronic pain, psychological distress, and medication burden interact to limit functional recovery. In practice, these elements could be bundled into a structured weekly programme combining two to three sessions of multicomponent exercise with one to two sessions of tai chi, qigong, or other mind–body practice, supplemented by tele-health-delivered CIH contact for symptom monitoring between in-person sessions.

## 7. A Multidimensional Integrative Framework: Implementation and Economics

### 7.1. Conceptualisation: BPEM Framework

The epistemological basis of the BPEM framework is pragmatic and systems-oriented rather than derived from a single causal theory. Evidence is organised along three linked axes: the domain of need addressed, the stage and resource level at which an intervention can be delivered, and the certainty and directness of the supporting evidence. Integration therefore refers to the explicit mapping of these axes, not to an assumption that all included modalities share a common mechanism or equivalent evidentiary status. Its original contribution lies in this mapping architecture and its application to war-related polytrauma in the Ukrainian context, whereas the component interventions and most underlying concepts are synthesised from previous literature.

The three tiers of the proposed framework are differentiated by injury severity, resource availability, and care setting, not by the type of intervention alone. The following criteria guide allocation:

○ Tier I (Acute and Intensive Care/Western Trauma Medicine) applies to patients with an Injury Severity Score (ISS) ≥ 16 or with life-threatening injuries to two or more body systems, who are in acute or post-surgical care settings. Primary goals aimed for are physiological stabilisation, pain control, wound management, and prevention of secondary complications. Rehabilitative goals at this tier are functional preservation rather than functional recovery. Tier I rehabilitation is embedded within, rather than separate from, the existing tertiary trauma and surgical care structure. Patients with complex polytrauma may require staged or repeated surgical management, including serial debridement, revision amputation, orthopaedic reconstruction, or management of wound- and implant-related complications, while early rehabilitation proceeds whenever medically feasible. The framework therefore assumes ongoing coordination and bidirectional referral between rehabilitation teams and tertiary centres capable of definitive specialist surgical management, rather than positioning rehabilitation as a strictly downstream phase of care.
○ Tier II (Sub-Acute and Specialist Rehabilitation/Chinese Intelligent Rehabilitation) applies to patients who are medically stable, have completed primary surgical management, and are engaged in structured inpatient or outpatient rehabilitation. This tier includes patients with ISS 9–15 or with significant single-system injuries such as TBI, limb loss, and spinal injuries requiring specialist input. Goals are functional recovery, prosthetic/assistive technology fitting, and commencement of psychological intervention.
○ Tier III (Community and Long-Term Maintenance /Eastern Traditional Medicine) applies to patients who have stopped improving or who are reintegrating into community or vocational roles. This tier includes all patients with chronic pain, PTSD, or ongoing functional limitation managed outside specialist centres. Non-pharmacological and integrative adjuncts are most applicable at this tier.

In essence, Tier I is to stabilise, Tier II is to rebuild, and Tier III is to sustain and integrate. A single patient may receive care across multiple tiers concurrently. For example, a patient in Tier I may simultaneously receive psychological first aid (Tier II/III), and tier assignment should be reviewed at each rehabilitation milestone. The allocation criteria above are clinical benchmarks for resource planning, not rigid exclusion criteria. A key exception applies to all economic analyses reviewed in this section. The data presented originate from the United States (VA Polytrauma System cost modelling), Canada (acupuncture ICER from primary care), and hypothetical Markov models [44,45,69,70]. For the Ukrainian healthcare cost structure, medical equipment procurement costs, insurance mechanisms, and the additional complexity of active wartime resource constraints have not been validated. At least two cost drivers are expected to differ materially from the source settings: wartime logistics and security overheads that raise equipment procurement and transport costs, and reimbursement structures that, unlike the insurance-based systems from which the cited figures derive, remain largely state-funded and still under reform in Ukraine. These figures are therefore cited to illustrate general cost-effectiveness directionality rather than to provide transferable Ukrainian budget impact estimates. Dedicated health-economic modelling adapted to Ukrainian unit costs and resource availability is a priority recommendation of this review.

The proposed multidimensional integrative framework is grounded in a biopsychosocial–existential and meaning-centred (BPEM) framework, which builds on Engel’s original biopsychosocial model to formally incorporate a fourth dimension, “existential and meaning-centred”. This domain is treated separately from the psychological and social domains rather than as a subcomponent of either, because its target constructs—moral injury, loss of meaning, and disrupted sense of purpose—are not consistently captured by standard psychological outcome measures (PTSD scales) or by social-support measures, and because the interventions that address it (Adaptive Disclosure Therapy, chaplaincy-based and meaning-centred approaches) operate on constructs distinct from mood regulation or social reintegration. Conceptually, the psychological domain primarily concerns symptoms, cognitions, and affect regulation; the social domain concerns relationships, roles, and participation; and the existential domain concerns moral appraisal, meaning, identity continuity, and purpose. These domains interact, but they are not interchangeable. Moral injury and loss of meaning may remain clinically salient even when PTSD symptoms or social support improve, which supports analysing this domain separately while retaining reciprocal links across all four domains [93,94,95,96]. A patient can achieve good psychological and social outcomes by conventional measures while remaining unable to make sense of what happened to them or to those around them; the existential domain is intended to capture that residual, clinically observed gap. Evidence from chronic pain populations also associates religiosity or spirituality with pain-related coping and quality of life [97,98], although these findings do not establish war-specific effectiveness. The “existential and meaning-centred” dimension may provide existential meaning-making structures that enable individuals to situate suffering within a larger framework of purpose, mobilise community and faith-based social support, and modulate the perceived uncontrollability of pain [97,98]. In combat populations presenting with moral injury, survivor guilt, and a profound loss of meaning, addressing existential needs plays a vital role in the recovery process. For example, a veteran who survived an incident in which fellow soldiers died may achieve good physical and functional recovery through standard rehabilitation while remaining unable to reintegrate socially because of unresolved guilt and a loss of sense of purpose; meaning-centred approaches, delivered alongside conventional psychotherapy, aim to help such patients situate the experience within a broader framework of meaning and renewed purpose, complementing rather than replacing standard care. This review would be the first to apply a three-tiered, resource-stratified implementation framework specifically to the Ukrainian war rehabilitation context, synthesising physical, technological, psychological, and existential-meaning-centred evidence domains within a unified operational model. The individual components of this model are not new in isolation: the biopsychosocial axis follows Engel’s original formulation, and tiered resource-stratified care pathways already exist in the NATO Role 4, UK Defence Medical Rehabilitation Centre, and U.S. VA Polytrauma models cited above. What this review contributes is the combination of three elements that, to our knowledge, have not been proposed together: (1) formal extension of the biopsychosocial model with a fourth, existential-meaning domain operationalised for combat-related moral injury; (2) explicit mapping of that four-domain structure onto a three-tier resource-stratified pathway; and (3) calibration of both to the specific injury profile, resource constraints, and cultural context of the Ukrainian conflict. The framework should therefore be read as an original organisational architecture built from established components, rather than as a set of newly discovered clinical mechanisms or an evidence-validated care model. Existing frameworks including the NATO Role 4 model, the UK Defence Medical Rehabilitation Centre tiered system, and the U.S. VA Polytrauma System of Care provide important structural precedents [20,62,63]. However, none were designed for the specific resource constraints, injury profile, and sociocultural context of the ongoing Ukrainian conflict.

It should be recognised that the BPEM extension, and specifically the “existential and meaning-centred” domain, is not yet consistently defined or operationalised within rehabilitation. Its expression and acceptable modes of care are culturally contingent, and its empirical literature is less developed than that supporting the biological and psychological domains. It is therefore proposed as a conceptually distinct but interacting domain for future operationalisation and testing, not as an evidence-validated clinical or implementation model.

Consistent evidence supports the centrality of psychosocial factors to rehabilitation outcomes. Across diverse chronic-pain populations, psychosocial variables including depression, anxiety, catastrophising, and social isolation consistently outperform objective pain intensity in predicting disability, healthcare utilisation, and poor treatment outcomes [13]. As a cognitive–affective triad comprising rumination, helplessness, and exaggeration, pain catastrophising routinely predicts a diminished response to pain killers and surgical and psychosocial treatments [13]. A rehabilitation model focused on the biological axis addresses less than half of the outcome predictors for the average war-injured patient.

### 7.2. Domain Mapping

**Figure 2:**
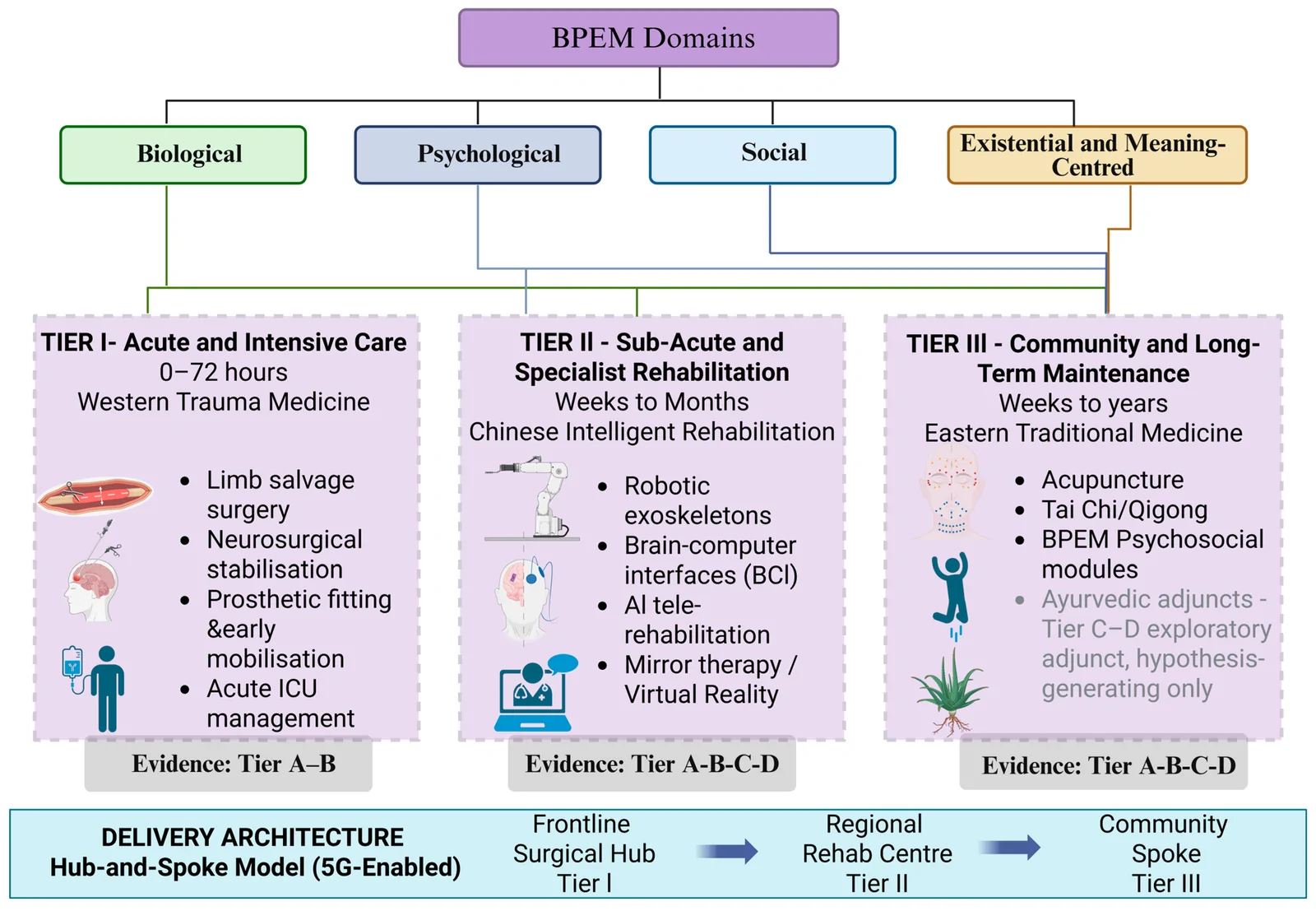
BPEM integrative rehabilitation framework for Ukrainian war-injured personnel. Interventions are stratified through three implementation tiers and four biopsychosocial–existential and meaning-centred (BPEM) domains. The “existential and meaning-centred” domain is a conceptual framework supported by emerging evidence and is not operationalised as a clinical recommendation [97,98,99].

By incorporating these four distinct domains, the proposed model broadens the scope of traditional rehabilitation. The biological domain is targeted by the integration between Western surgical stabilisation and technology-based rehabilitation such as robotic-exoskeleton-assisted gait training on one hand and BCI-based neuroplasticity exercises and acupuncture on the other. Peripheral pathway mediations, spinal GABAergic mechanisms, and supraspinal endorphin effects provide strong neurobiological validation for the use of acupuncture in pain modulation. The psychological domain is addressed by psychosocially integrated programmes such as the Invincibility Programme, mind–body approaches of tai chi and integrated exercise, and psychologically integrated physiotherapy that includes graded activity as well as cognitive–behavioural and acceptance-based approaches [99]. The social domain is addressed by involving families in rehabilitation, peer support, interpersonal CIH, and occupational retraining. The fourth domain of the framework, here termed “existential and meaning-centred” rather than “spiritual” to reflect its clinical operationalisation, encompasses the treatment of moral injury, survivor guilt, grief, and existential distress. These constructs may overlap with PTSD or depression but are not reducible to symptom severity or social participation alone [93,94]. This domain is operationalised clinically through validated instruments including the Moral Injury Symptom Scale (MISS) and the Moral Injury Events Scale (MIES), and through manualised treatment protocols that have progressed to RCT evaluation, including Adaptive Disclosure Therapy (AD-MIL), Trauma-Informed Guilt Reduction Therapy (TrIGR), and Building Spiritual Strength [94,95,96]. In low-resource settings, military chaplaincy and community peer-support programmes represent accessible delivery mechanisms. This domain is graded Tier B for moral-injury-specific psychotherapy in veteran populations, and Tier C for operational implementation in Ukrainian frontline-adjacent settings where specialist mental health resource constraints apply. The complete three-tiered implementation pathway, including the BPEM domain assignments, evidence grades, and delivery architecture for each pillar, is presented in Figure 2.

### 7.3. Cross-Cultural and Regulatory Considerations

The proposed framework requires regulatory, cultural, standardisation, and technology-deployment conditions to support implementation. Acupuncture, Ayurvedic medicine, and technology-based rehabilitation require temporary practice guidelines, competency standards for traditional medicine practitioners, and evidence-based registration of herbal medicines [1]. The regulatory approaches outlined in the World Health Organisation (WHO) Traditional Medicine Strategy 2019–2025, combined with the ongoing development of Ukraine’s national rehabilitation strategy, offer a clear pathway for structural support [1].

Effective clinical integration requires continuous cultural adaptation that moves beyond simple translation. Ukrainian patients with sufficient cultural mediation in Norwegian MEDEVAC facilities experienced satisfactory rehabilitation [8], suggesting that theoretical perspective differences can be addressed. Acupuncture and mind–body programs designed for Ukraine’s military will face Soviet legacies of passive and pharmacologically oriented care. Ukrainian practitioners of integrative methods will need to work as cultural brokers. Safety protocols, such as peer support, trauma-sensitive care, and Ukrainian-language therapeutic resources, should be put in place first to enable the successful implementation of concurrent interventions.

Experience from tiered tele-medicine networks in geographically remote settings suggests that a hub-and-spoke structure may improve access to specialist input [28]. However, transfer to Ukraine would require adaptation to wartime connectivity, workforce, security, maintenance, and regulatory constraints. Implementation should therefore proceed incrementally, prioritising workforce preparation and reliable low-bandwidth service delivery before introducing resource-intensive robotic or AI-supported systems.

### 7.4. Economic Analysis

International modelling and cohort evidence suggests that technology-assisted rehabilitation, acupuncture, and early specialist multidisciplinary rehabilitation may reduce long-term costs through improved functional independence, lower care requirements, and return to employment [20,44,45,69,70]. However, these estimates are derived from non-Ukrainian settings and cannot be transferred directly because utilisation rates, wartime logistics, equipment procurement, workforce costs, and reimbursement structures differ substantially. The priority should therefore be the development of a locally calibrated decision-analytic model incorporating Ukrainian service costs, implementation constraints, and relevant health-outcome measures before firm claims of cost-effectiveness are made.

## 8. Discussion

The central implication of this review is that war-related polytrauma rehabilitation should be approached as a multidomain recovery process rather than as a sequence of isolated physical, neurological, psychological, and social interventions. Across the evidence reviewed, the major determinant of sustained recovery is not the effectiveness of any single modality alone, but the extent to which rehabilitation systems can interrupt reinforcing cycles among pain, impaired mobility, psychological distress, and delayed social reintegration. The proposed framework therefore emphasises feedback relationships across domains of recovery. Physical recovery is shaped not only by the intensity and quality of rehabilitation therapy, but also by pain control, psychological readiness, cognitive function, family support, and opportunities for vocational or community reintegration. Conversely, persistent pain, PTSD symptoms, reduced mobility, and social withdrawal may reinforce one another and limit functional gains even when technically appropriate rehabilitation is available. Technology-assisted interventions are best understood as access enhancers and intensity multipliers, whereas non-pharmacological adjuncts primarily support symptom modulation, engagement, and medication burden reduction. Their value lies in how they are layered into an integrated rehabilitation pathway, rather than in their use as stand-alone substitutes for core rehabilitation. A systematic comparative analysis of the proposed framework against four established international military rehabilitation models is presented in Table S2 [1,8,15,28,29].

To distinguish evidentiary roles, Tier A–B findings are used to identify interventions with comparatively stronger support within their source populations, whereas Tier C–D findings are included only as complementary or hypothesis-generating options. Lower-tier interventions are not presented as standards of care, and evidence derived from non-war populations does not establish war-specific effectiveness. The following implementation guidance should be read as derived from the individual evidence bases for each intervention rather than from validation of the BPEM framework as a whole, which remains a conceptual proposal pending empirical testing. This interpretation has practical implications for clinical investment and future research. Technology-assisted and adjunctive therapies should be evaluated according to their position within the rehabilitation system: whether they increase therapeutic dose, expand access, improve adherence, reduce pain or psychological burden, or support reintegration. Robotic exoskeletons are best justified clinically and economically when focused on people with complete and near-complete neurological injury, and in the early phase of rehabilitation. Their value may be more selective in incomplete injuries or later chronic phases, where treatment goals, patient selection, and cost-effectiveness assumptions differ. BCIs enhance traditional therapy and require at least four weeks of delivery to be effective. AI tele-rehabilitation requires interactivity and real-time feedback; passive, asynchronous delivery offers minimal additional benefit. Acupuncture is Tier A for neuropathic pain and Tier B for cost-effectiveness, while PLP is Tier C–D and, consistent with its exploratory evidence base, should be considered hypothesis-generating rather than a basis for routine clinical adoption pending dedicated trials. Ayurvedic therapies are similarly Tier C–D adjuncts, presented here as candidates for staged evaluation rather than as validated interventions, requiring standardised preparation, safety monitoring, and further study before broader clinical implementation. For Ukraine, the major challenge is translating available evidence into feasible service delivery under wartime constraints. The structural barriers highlighted throughout this review spanning workforce capacity, regulatory frameworks, technology deployment, and cultural–epistemological differences closely mirror challenges previously encountered in global healthcare integration. The key resource required is not bricks and mortar, but rather new clinical culture and training. The risk of opioid dependency is a clinical and policy issue that requires the involvement of non-opioid pain management as an integral part of the rehabilitation process.

### Limitations

Several interpretive considerations should guide the application of this synthesis. The evidence base spans heterogeneous populations, intervention types, and outcome measures, which makes quantitative pooling inappropriate and places greater importance on clinically oriented interpretation. Some of the strongest technology-assisted rehabilitation evidence derives from stroke, SCI, chronic pain, or general veteran populations rather than directly from war-related polytrauma cohorts. These data remain relevant because they address core impairments also observed after combat injury, but their application to Ukrainian war-injured patients, who often present with complex multimorbidity (concurrent TBI, amputation, PTSD, chronic pain, and social disruption), requires careful adaptation, and the strength of any clinical-effectiveness claims drawn from this evidence should be interpreted cautiously rather than assumed to be directly generalisable. Similarly, Ukraine provides a high-priority case study of large-scale wartime rehabilitation need, but implementation models will require contextual adjustment in other conflict-affected health systems. Future studies should therefore prioritise war-specific rehabilitation cohorts, pragmatic implementation trials, long-term functional outcomes, opioid-sparing endpoints, and health-economic modelling using local cost structures.

## 9. Conclusions

After combat injury, multidimensional approaches to rehabilitation may be beneficial and go beyond biomedical approaches. The combination of biomedical rehabilitation, technology-assisted rehabilitation, and non-pharmacological additional treatments may serve as an integrated, layered approach to address the different areas of human function affected by combat injury, rather than stand-alone alternatives. Within this layered model, biomedical rehabilitation provides physiological stabilisation and early functional preservation; technology-assisted rehabilitation may increase therapeutic intensity, access, and neuroplastic engagement; non-pharmacological adjuncts may contribute to pain control, psychological regulation, and medication-burden reduction; and psychosocial and meaning-centred care may support the integration of functional gains into sustained recovery and social reintegration.

Beyond treatment efficacy, the proposed model identifies economic hypotheses relevant to resource-constrained healthcare systems. International evidence suggests that robotic systems may approach cost-neutrality with sufficient utilisation, acupuncture may be cost-effective in selected non-war settings, and early multidisciplinary rehabilitation may generate downstream savings that offset part of the initial programme costs. These estimates require local validation before they can support Ukrainian implementation or budget decisions.

Four areas of research and implementation are proposed to bring this about. First, culturally sensitive robust RCTs should be carried out to investigate acupuncture for PLP in Ukrainian veterans and multidisciplinary BPEM rehabilitation vs. standard care in Ukrainian rehabilitation centres. Second, feasibility trials should test 5G robotic rehabilitation in remote or front-line-adjacent rehabilitation facilities. Third, a health-economic model should be developed for Ukraine that incorporates cost-effectiveness estimates from the international literature, with local costs and DALY weights. Fourth, the primary implementation cost should be dedicated to workforce training, focusing on psychologically informed practice, BPEM clinical reasoning, and the safe practice of integrative modalities.

The current evidence base provides preliminary support for a multidimensional integrative approach to war-related rehabilitation. The BPEM architecture should therefore be used as a conceptual guide to structure future empirical testing and context-specific adaptation, not as a validated implementation model. To begin to harness this potential, war-specific clinical evidence, context-specific implementation strategies and lifelong capacity building in rehabilitation services are required.

## Data Availability

No new data were created or analysed in this study. Data sharing is not applicable to this article.

## Supplementary Materials

The following supporting information can be downloaded at: https://www.mdpi.com/article/10.3390/healthcare14172778/s1, Table S1: Correlation Between Ukraine War-Related Injury Profiles and Targeted Rehabilitation Interventions with Evidence Grades and Tier Priorities; Table S2: Comparative Analysis of International Military Rehabilitation Frameworks Against the Proposed BPEM Tri-System Model; Table S3: Botanical Profiles, Mechanisms of Action, and Clinical Evidence for Ayurvedic and Eastern Herbal Agents Proposed in the Tri-System Framework.

## Abbreviations

The following abbreviations are used in this manuscript:

AI	Artificial Intelligence
APR	Acute Polytrauma Rehabilitation
ASD	Acute Stress Disorder
ASIC3	Acid-Sensing Ion Channel 3
ATP	Adenosine Triphosphate
BCI	Brain–Computer Interface
BPEM	Biopsychosocial–Existential and Meaning-Centred
CDC	Centre for Disease Control
CIH	Complementary and Integrative Health
CPTSD	Complex Post-Traumatic Stress Disorder
CSQ-8	Client Satisfaction Questionnaire-8
CT	Computed Tomography
DALY	Disability-Adjusted Life Year
DoD	Department of Defense
EEG	Electroencephalography
GMP	Good Manufacturing Practice
ICER	Incremental Cost-Effectiveness Ratio
IETP	Intensive Evaluation and Treatment Programme
IL-6	Interleukin 6
MI	Motor Imagery
NF-κB	Nuclear Factor Kappa B
PLP	Phantom Limb Pain
PTRP	Polytrauma Transitional Rehabilitation Programme
PTSD	Post-Traumatic Stress Disorder
QALY	Quality-Adjusted Life Year
RCT	Randomised Controlled Trial
RLP	Residual Limb Pain
SCI	Spinal Cord Injury
sEMG	Surface Electromyography
TBI	Traumatic Brain Injury
TCM	Traditional Chinese Medicine
TNF-α	Tumour Necrosis Factor Alpha
TRP	Transient Receptor Potential
VA	Veterans Affairs
WHO	World Health Organisation
