# Supplementary material for: Integrative Rehabilitation for War-Related Polytrauma: A Narrative Review of Multidimensional Strategies and Implementation Challenges
Source: Healthcare (Basel). 2026 Sep 1;14(17):2778. doi: 10.3390/healthcare14172778 (PMC13565737; doi:10.3390/healthcare14172778)
Supplement: Supplementary file 1 [file healthcare-14-02778-s001.zip › healthcare-4406537-supplementary.pdf]

Integrative Rehabilitation for War-Related Polytrauma: A Narrative Review of Multidimensional Strategies and Implementation Challenges

Supplementary Data

Table S1. Correlation Between Ukraine War-Related Injury Profiles and Targeted Rehabilitation Interventions with Evidence Grades and Tier Priorities

Table S1 presents a structured evidence matrix mapping each proposed rehabilitation intervention to the eight primary injury categories identified in the Ukrainian conflict, graded by the highest available level of clinical evidence. The programs are further sorted based on their delivery tier within the proposed BPSS Tri-System Framework: Tier-I (Acute Phase, high-evidence priorities), Tier-II (Post-Acute Phase, moderate-to-high evidence), and Tier-III (Community / Specialised Phase, emerging or case-report evidence) [1,2,4,6,15,17,22-25,33-37,42-45,50,51,54-57,58,61-67,69-73].

Intervention	Tier	Blast / Polytrauma	Amputation / PLP	SCI / Spinal	TBI / Brain	PTSD / ASD	Neuropathic Pain	Vestibular / Balance	Opioid Reduction
**TIER I - High Evidence, Immediate Priority (Acute Phase)**									
**Acute surgical salvage & wound management**	I	A	A	-	-	-	-	-	-
**Prosthetic fitting & early mobilisation**		-	A	-	-	-	B	-	-
**Neuro-stabilisation (TBI, spinal fractures)**		B	-	B	A	-	-	-	-
**TIER II - Moderate-High Evidence, Post-Acute Priority**									
**Robotic exoskeleton training**	II	A	A	A	B	-	-	-	B
**AI telerehabilitation**		B	B	B	B	B	A	-	B
**BCI (MI-BCI)**		-	B	B	B	-	-	-	-

Intervention	Tier	Blast / Polytrauma	Amputation / PLP	SCI / Spinal	TBI / Brain	PTSD / ASD	Neuropathic Pain	Vestibular / Balance	Opioid Reduction
**Mirror therapy / Virtual reality**		-	B	-	-	-	-	-	-
**Invincibility Program / ACT / Psychosocial**		-	-	-	B	B	-	-	B
**Psychologically informed physiotherapy**		B	-	B	B	A	B	-	-
**TIER III - Emerging Evidence, Community / Specialised Phase**									
**Panchakarma / Ayurvedic protocols**	III	D	D	-	-	-	D	-	-
**Acupuncture (EA / MA)**	III	-	B	-	-	B	A	-	A
**Tai Chi / Qigong**	III	-	-	-	-	B	-	A	-

Intervention	Tier	Blast / Polytrauma	Amputation / PLP	SCI / Spinal	TBI / Brain	PTSD / ASD	Neuropathic Pain	Vestibular / Balance	Opioid Reduction
**Osseointegration + robotic interface**		-	D	-	-	-	-	-	-
**Tai Chi remote (RCT protocol)**		-	-	-	-	D	-	D	-

Evidence: A = randomised controlled trials and meta-analyses (robust evidence); B = observational cohort studies (moderate evidence); C = descriptive and qualitative studies (moderate/emerging evidence); D = emerging or exploratory evidence (hypothesis-generating). - = not applicable or no evidence identified in this population. Tier classification reflects implementation priority within the proposed BPSS Tri-System Framework (see Figure 2).

Table S2. Comparative Analysis of International Military Rehabilitation Frameworks Against the Proposed BPEM Tri-System Model

Table S2 compares four established international military rehabilitation models the U.S. Veterans Affairs (VA) Polytrauma System of Care, NATO Role 4, the IDF Model, and the UK Major Trauma Network APR Model against the proposed BPSS Tri-System Framework on four dimensions: primary focus, key strength, key limitation in active or dispersed conflict, and the specific innovation contributed by the Tri-System approach [1,8,15,28,29].

Framework / Model	Primary Focus	Key Strength	Limitation in Active / Dispersed Conflict Settings	Tri-System Innovation Point
**U.S. Veterans Affairs (VA) Polytrauma System of Care**	Patient-centred complete care such as integrates complementary therapies (yoga and acupuncture) alongside conventional rehabilitation	Strong institutional infrastructure; strong biopsychosocial integration; well-funded interdisciplinary teams	Designed for a stable, high-resource domestic setting; requires specialist personnel density unavailable in active conflict zones	The Tri-System Framework operationalises integrative care for frontline and remote settings via a 5G-enabled hub-and-spoke model

Framework / Model	Primary Focus	Key Strength	Limitation in Active / Dispersed Conflict Settings	Tri-System Innovation Point
**NATO Role 4 Rehabilitation Framework**	Echelon-based medical logistics. Standardised evacuation from Role 1 to Role 4 (point of injury to conclusive care)	Highly standardized and efficient to ensures conclusive care reaches all injured personnel regardless of location	Primarily biomedical and logistical. lacks a formal mind-body or spiritual pillar and does not address long-term community reintegration	Bridges the gap between high-level acute surgery and long-term neuroplastic or spiritual recovery through Pillars II and III
**Israel Defense Forces (IDF) Model**	Rapid transition to community rehabilitation. peer-support and social reintegration as primary recovery tools	World-class social reintegration outcomes; strong peer-support networks and culturally integrated psychosocial care	Geography-dependent (small landmass with centralised facilities) and not scalable across large, dispersed conflict theaters like Ukraine	Spreads out community care through AI-telerehabilitation and modular robotics, enabling peer-level outcomes at scale
**UK Major Trauma**	Acute Polytrauma Rehabilitation (APR) within 24-72 hours of injury; three-	Demonstrated net lifetime savings of ~£500,000 per severely injured patient;	Resource-intensive; delayed care transitions and specialist shortages replicate the	Supplements the APR model with a non-pharmacological pain management pillar (TCM

Framework / Model	Primary Focus	Key Strength	Limitation in Active / Dispersed Conflict Settings	Tri-System Innovation Point
**Network (APR Model)**	stages (acute, post-acute, community)	return-to-work rates of 37-82%	bottlenecks Ukraine currently faces	/ Ayurveda), reducing opioid dependency risk
**Proposed BPSS Tri-System Framework**	BPSS integration of Western surgery, Chinese intelligent rehabilitation, and Eastern traditional medicine across all four BPSS domains	First framework to treat robotics and Eastern medicine as equal system-level partners; explicitly addresses the spiritual / existential dimension of combat injury; tiered evidence system (Tier-I to III) provides implementable priorities	Currently theoretical; requires empirical validation in conflict-specific settings; Ayurvedic components are hypothesis-generating only	**Primary contribution: unified tri-system BPSS architecture scalable for resource-constrained, geographically dispersed conflict environments**

Table S3. Botanical Profiles, Mechanisms of Action, and Clinical Evidence for Ayurvedic and Eastern Herbal Agents Proposed in the Tri-System Framework

Table S3 provides detailed botanical and pharmacological profiles for seven herbal agents and formulations from the Eastern traditional medicine pillar of the proposed framework. Profiles include mechanistic pathway data, rehabilitation application, evidence grade, key supporting references, and safety considerations relevant to deployment in Ukrainian clinical settings. These agents are classified as **Tier-III (Tertiary) hypothesis-generating measures** and are not recommended for immediate clinical implementation without further controlled investigation. All preparations are required certified GMP-compliant supply chains and systematic pharmacovigilance prior to use. Evidence grades follow the same system that has used in Table S I [71–79].

Herb / Agent	Origin / Tradition	Mechanism of Action	Rehabilitation Application	Evidence Grade & Clinical Data	Key References & Safety Notes
**Curcumin** ***(Curcuma longa)***	Turmeric rhizome; Indian	Inhibits NF-κB, COX-2, and MAPK	Systemic inflammation from blast polytrauma	Grade A - meta-analysis of 11 RCTs (n=1,009) confirms pain relief and	[78, 72] GI-safe and CYP450 interaction with

Herb / Agent	Origin / Tradition	Mechanism of Action	Rehabilitation Application	Evidence Grade & Clinical Data	Key References & Safety Notes
	/ Sri Lankan Ayurveda	pathways; reduces TNF-α, IL-1β, IL-6	joint pain and Opioid-reducing supplement	functional improvement superior to placebo, non-inferior to NSAIDs with better GI safety profile	anticoagulants pre-treatment screening required
**Boswellic acids *(Boswellia serrata)***	Indian frankincense resin and Traditional Indian medicine	Inhibits 5-lipoxygenase; suppresses NF-κB; reduces leukotriene-mediated inflammation	Neuropathic pain; post-traumatic arthropathy; synovial inflammation following extremity polytrauma	Grade A - same Bannuru et al. meta-analysis; boswellia significantly superior to control treatment for pain and functional outcomes	[78, 72] Caution with anticoagulants (Boswellia increases bleeding time); monitor INR
**Withanolides *(Withania***	Ashwagandha root and Indian	Modulates HPA axis, reduces cortisol, anti-	Psychological resilience, PTSD	Grade B - pilot double-blind placebo-controlled	[72, 77]

Herb / Agent	Origin / Tradition	Mechanism of Action	Rehabilitation Application	Evidence Grade & Clinical Data	Key References & Safety Notes
somnifera / Ashwagandha)	Ayurveda (Rasayana class)	inflammatory via NF-κB and MAPK suppression; immunomodulatory	symptom modulation, fatigue and immune reconstitution post-trauma	RCT shows enhanced immune function in adults; immunomodulatory pathway data from Upadhyay et al.	Caution with immunosuppressants; avoid in thyroid disorders; no hepatotoxic risk at standard doses
**Guggulu *(Commiphora mukul)***	Oleo-gum resin; Sri Lankan & Indian Ayurveda	Anti-inflammatory and hypolipidaemic; guggulsterones modulate NF-κB and thyroid activity	Joint stiffness, post-traumatic arthropathy and inflammation metabolite clearance via Panchakarma Basti	Grade D - clinical evidence from Kizhakkeveettil et al. case report: Katibasti with Guggulu formulations improved ODI 55% to 20% and	[73]<br>Interaction with anticoagulants and thyroid medications, requires pharmacovigilance

Herb / Agent	Origin / Tradition	Mechanism of Action	Rehabilitation Application	Evidence Grade & Clinical Data	Key References & Safety Notes
				pain 9-10/10 to 0-2/10 over 8 weeks	
***Mahanarayana Tailam* (polyherbal oil)**	Classical Ayurvedic medicated oil; Sri Lankan Deshiya Chikitsa	Topical anti-inflammatory and analgesic; penetrates via transdermal lipid pathways; modulates COX-2 locally	Katibasti procedure for musculoskeletal pain, joint mobility and spinal injury rehabilitation	Grade D - case report evidence only, used as part of the Kizhakkeveettil et al. protocol and no standalone RCT data available	[73, 74] Topical application - low systemic absorption; certified GMP-grade preparation required per De Silva protocol
***Dashamoola decoction* (ten-root formulation)**	Classical ten-root herbal blend and Ayurveda	Analgesic, anti-inflammatory, and antioxidant via combined	Systemic inflammation, nerve pain, as base for Yapana Basti and	Grade D - clinical use within Kizhakkeveettil et al. Panchakarma protocol; also referenced	[73] Complex poly-herbal formulation; requires

Herb / Agent	Origin / Tradition	Mechanism of Action	Rehabilitation Application	Evidence Grade & Clinical Data	Key References & Safety Notes
	(Basti therapy base)	phytochemical pathways; supports Vata-pacifying action	musculoskeletal rehabilitation in polytrauma	in autoimmune polymyositis case data	standardised preparation and pre-treatment herb-drug interaction screening
**Sri Lankan ethnobotanicals *(Coriandrum sativum, Coscinium fenestratum, Adhatoda vasica et al.)***	Gampaha District, Western Province, Sri Lanka	Modulate TNF-α, IL-6, COX-2, and 5-lipoxygenase via aqueous infusions, poultices, and medicated oils	Locally available anti-inflammatory adjuncts; feasible in field-adjacent settings; support Panchakarma externally applied phases (Abhyanga, Shirodhara)	Grade C - ethnobotanical study of 43 species with highest inter-informant consensus; not yet validated in RCTs; feasibility in conflict zones is theoretical	[71]<br>Requires species verification; risk of misidentification in field conditions; certified supply chain is mandatory prerequisite

Herb / Agent	Origin / Tradition	Mechanism of Action	Rehabilitation Application	Evidence Grade & Clinical Data	Key References & Safety Notes
Herb-drug interaction categories: HIGH RISK (anticoagulants, immunosuppressants - require pre-treatment screening); MODERATE (thyroid medications, CYP450 substrates monitor); LOW (topically applied modalities). GMP = Good Manufacturing Practice. ODI = Oswestry Disability Index. All Tier-III herbal agents are required certified supplier chains and systematic pharmacovigilance before clinical deployment in Ukrainian settings.					
